# Artificial Seawater Media Facilitate Cultivating Members of the Microbial Majority from the Gulf of Mexico

**DOI:** 10.1128/mSphere.00028-16

**Published:** 2016-04-27

**Authors:** Michael W. Henson, David M. Pitre, Jessica Lee Weckhorst, V. Celeste Lanclos, Austen T. Webber, J. Cameron Thrash

**Affiliations:** Department of Biological Sciences, Louisiana State University, Baton Rouge, Louisiana, USA; University of British Columbia

**Keywords:** Gulf of Mexico, SAR11, artificial seawater, coastal microbiology, high-throughput culturing, marine microbiology

## Abstract

High-throughput cultivation studies have been successful at bringing numerous important marine bacterioplankton lineages into culture, yet these frequently utilize natural seawater media that can hamper portability, reproducibility, and downstream characterization efforts. Here we report the results of seven experiments with a set of newly developed artificial seawater media and evaluation of cultivation success via comparison with community sequencing data from the inocula. Eighty-two new isolates represent highly important marine clades, including SAR116, OM60/NOR5, SAR92, *Roseobacter*, and SAR11. For many, isolation with an artificial seawater medium is unprecedented, and several organisms are also the first of their type from the Gulf of Mexico. Community analysis revealed that many isolates were among the 20 most abundant organisms in their source inoculum. This method will expand the accessibility of bacterioplankton cultivation experiments and improve repeatability by avoiding normal compositional changes in natural seawater.

**IMPORTANCE** The difficulty in cultivating many microbial taxa vexes researchers intent on understanding the contributions of these organisms to natural systems, particularly when these organisms are numerically abundant, and many cultivation attempts recover only rare taxa. Efforts to improve this conundrum with marine bacterioplankton have been successful with natural seawater media, but that approach suffers from a number of drawbacks and there have been no comparable artificial alternatives created in the laboratory. This work demonstrates that a newly developed suite of artificial-seawater media can successfully cultivate many of the most abundant taxa from seawater samples and many taxa previously only cultivated with natural-seawater media. This methodology therefore significantly simplifies efforts to cultivate bacterioplankton and greatly improves our ability to perform physiological characterization of cultures postisolation.

## Abstract (precis)

The difficulty in cultivating many microbial taxa vexes researchers intent on understanding the contributions of these organisms to natural systems, particularly when these organisms are numerically abundant, and many cultivation attempts recover only rare taxa. Efforts to improve this conundrum with marine bacterioplankton have been successful with natural seawater media, but that approach suffers from a number of drawbacks and there have been no comparable artificial alternatives created in the laboratory. This work demonstrates that a newly developed suite of artificial seawater media can successfully cultivate many of the most abundant taxa from seawater samples and many taxa previously only cultivated with natural seawater media. This methodology therefore significantly simplifies efforts to cultivate bacterioplankton and greatly improves our ability to perform physiological characterization of cultures postisolation.

## INTRODUCTION

The study of microorganisms and their roles in remediation and biogeochemical cycling requires the observation of microbial communities and genetics in nature coupled with experimental testing of hypotheses both *in situ* and in laboratory settings. The latter is best accomplished by cultivation of microorganisms, yet the majority of the microorganisms observed under a microscope are not readily cultivated (1–3). High-throughput, dilution-to-extinction culturing (HTC) with natural seawater has been responsible for the isolation of strains representing numerically abundant clades such as SAR11 (4, 5), SUP05/Arctic96BD-19 (6), OM43 (7), a small-genome *Roseobacter* strain (8), and others. HTC benefitted from the oligotrophy theory and the research of Don Button and colleagues (9, 10), which demonstrated that because marine systems are frequently in a state of nutrient limitation, adaptation by the microbial denizens has led to unusual genomics and physiology that must be accounted for in cultivation strategies. These oligotrophic microorganisms are generally small, with streamlined genomes that eschew many complicated regulatory systems (11), resulting in slow growth and complicated nutrient requirements, including poor or no growth at high carbon concentrations (4, 7, 9, 10, 12). Thus, successful HTC experiments have provided a noncompetitive growth environment with naturally occurring compounds at *in situ* concentrations.

Until now, this has been accomplished predominantly via filtration and autoclaving of natural seawater by using protocols (9, 10, 13) that avoided excessive nutrients found in “traditional” artificial marine media (14) (see Table 3) and provided complex, but largely unknown, natural dissolved organic matter (DOM) components. However, in spite of its success, there are many drawbacks to using a natural seawater medium. First, it requires regular access to large volumes (20 liters or more) of seawater, which can be a major logistical hurdle for research labs not located near a coastal source and/or without vessel access. Second, natural seawater is inherently complex and undefined, thus limiting the amount of physiological characterization that can be accomplished postisolation (e.g., salinity growth optima or single-carbon substrate utilization). Third, seawater at a given location may experience significant intra- and interannual chemical fluxes, thereby creating “vintages” from specific sample collections that can prevent reproducible growth or repeated transfers when attempting to passage an organism from one vintage to another. We therefore developed a complex yet defined low-nutrient set of artificial seawater media (ASM) incorporating the theory of Button and colleagues and published measurements for marine systems, including the Gulf of Mexico, that account for variable salinity in coastal ecosystems (12, 15–18) (see Table S1 in the supplemental material; all supplemental material for this article may be found at https://thethrashlab.com/publications/).

## RESULTS AND DISCUSSION

To create the JW1 medium (Table 1; see Table 3), the basic salt mixture was derived by using values in the medium designed by Kester et al. (19). Concentrations of phosphate, iron, and trace metals were taken from a defined medium for SAR11 (12), with modified vitamin concentrations based on data in references 20 and 21. DOM is one of the most important yet least understood components of natural seawater, having varied carbon and nitrogen constituents that can be difficult to distinguish (22). A complex but defined list of carbon and nitrogen compounds was used to generate our modular DOM mixtures (see Table S1 in the supplemental material), and the total carbon and nitrogen concentrations were based on previously reported Gulf of Mexico data (16–18). Ultimately, our modular recipe allows for customization of the different carbon and nitrogen components, making it easily adaptable to a variety of environments.

**TABLE 1:** JW1 medium components

Component	Concn
Basic components	
Cl	0.54 M
Na	0.48 M
Mg	52 mM
S (SO_4_^2−^)	30 mM
Ca	10 mM
K	10 mM
Br	0.8 mM
B	0.42 mM
Sr	0.09 mM
F	0.055 mM
Fe	101 nM
P (PO_4_^3−^)	51 µM
HCO_3_^−^	10 mM
Total inorganic N^a^	45 µM
Total organic N^b^	23 µM
Total organic C^c^	71 µM
Trace metals	
Mn	9 nM
Zn	1 nM
Co	0.5 nM
Mo	0.3 nM
Se	1 nM
Ni	1 nM
Vitamins	
B_1_/thiamine	500 nM
B_2_/riboflavin	0.7 nM
B_3_/niacin	800 nM
B_5_/pantothenate	425 nM
B_6_/pyridoxine	500 nM
B_7_/biotin	4 nM
B_9_/folic acid	4 nM
B_12_	0.7 nM
*myo*-Inositol	500 nM
4-Aminobenzoic acid	60 nM

a As NO_3_^−^, NO_2_^−^, and NH_4_^+^.

b As urea and amino acids.

c As amino acids, carboxylic acids, sugars, and fatty acids.

**FIG 1:**
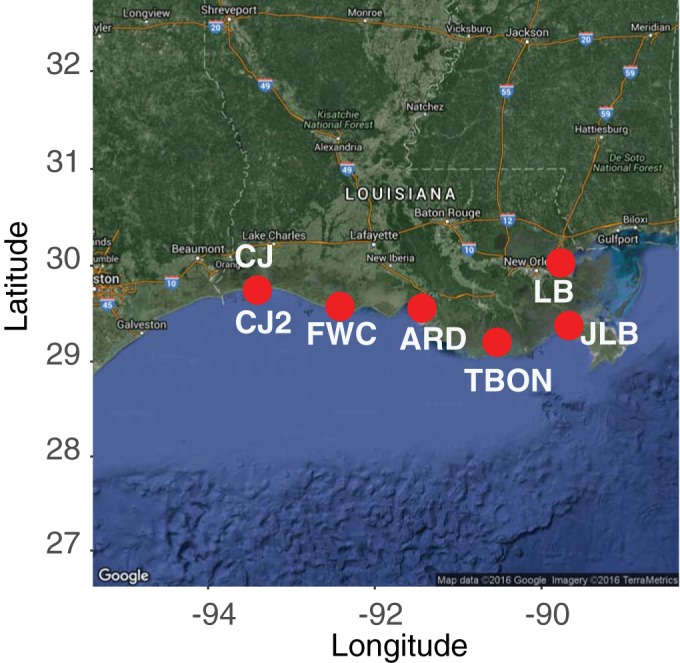
Locations of the seven experiments along the coast of the northern Gulf of Mexico (map data copyright 2016 Google; imagery copyright 2016 TerraMetrics).

Using the media in Table S1 in the supplemental material, we conducted seven HTC experiments with a combined total of 3,360 distinct cultivation wells according to the protocol in reference 15 and included community characterization (via 16S rRNA gene tag sequencing) of the source water for each experiment. These experiments used water collected from six sites along the southern Louisiana coastline (Fig. 1) that represent varied estuarine-marine systems (23). Our goals in sampling these different sites were to provide varied source inocula and to simultaneously collect coastal northern Gulf of Mexico microbial biogeography data. Initial cultivability (10) ranged from 0 to 53.1% (Table 2), and 82 of the 231 positive cultures across all of the experiments were capable of repeated transfer after initial exchange from polytetrafluoroethylene (PTFE) to polycarbonate flasks and/or did not contain multiple organisms. The 82 isolates represent various marine clades of *Gammaproteobacteria*, *Betaproteobacteria*, *Alphaproteobacteria*, and *Actinobacteria* (see Table S1 and Fig. S1 to S3 in the supplemental material), including five isolates from the *Roseobacter* clade, 23 isolates from the so-called “oligotrophic marine *Gammaproteobacteria*” (24), two isolates from clade OM43, and most notably the first two reported isolates of SAR11 and SAR116 from the Gulf of Mexico (see Table S1). The maximum observed cell yields of cultures grown in the JW1 to JW4 media generally ranged between 10^5^ and 10^6^ cells·ml^−1^, as determined by flow cytometry, with growth rates spanning a wide range (see Fig. S4 in the supplemental material).

**TABLE 2:** Initial cultivability statistics and salinity values for seven HTC experiments

Site	Date	No. of wells:		P	X	V^a^	% Cultivability	Medium	Medium salinity	Source salinity
		Inoculated	Positive							
CJ	9/12/14	460	15	0.033	1.27	0.026	2.6	JWAMPFe	34.8	24.6
ARD	11/24/14	460	1	0.002	1.5	0.001	0.1	JW1	34.8	1.7
JLB	1/9/15	460	61	0.133	1.96	0.073	7.3	JW1	34.8	26
FWC^b^	3/21/15	460	301	0.654	2	0.531	53.1	JW4	5.8	5.4
LB	6/9/15	460	0	0	1.8	0	0	JW4	5.8	1.4
Tbon	7/24/15	460	41	0.089	1.56	0.06	6	JW3	11.6	14.2
CJ2	10/1/15	460	61	0.133	2	0.071	7.1	JW2	23.2	22.2

a According to *V* = −ln(1 − *p*)/*X*, where *p* is the number of positive wells divided by the number of inoculated wells and *X* is the number cells inoculated per well (10). Percent cultivability = *V* × 100.

b Sample was counted by microscopy and believed to have underestimated the total number of cells, resulting in a higher percent cultivability.

Phylogenetic inspection shows that the ASM provided for cultivation success similar to that provided for by natural seawater media, as evidenced by the numerous close relationships to cultivars obtained with natural seawater media (designated with HTCC, HIMB, and IMCC) (see Fig. S1 to S3 and Table S1 in the supplemental material). LSUCC0245 is only the second isolate from SAR11 subclade Va, and LSUCC0261 represents only the third isolate from SAR11 subclade IIIa (25, 26). Two clades were notable for their phylogenetic novelty in the *Gammaproteobacteria*—those containing LSUCC0096 and LSUCC0101 (see Fig. S1). The clade containing LSUCC0096 was affiliated with a sole isolate, HIMB30, of the OM252 clade, and therefore, Louisiana State University Culture Collection (LSUCC) isolates represent a significant expansion of cultured representatives of this group. Similarly, LSUCC0101-type organisms matched best to an only recently recovered isolate, IMCC14953 (27), and this clade likely represents a novel gammaproteobacterial family on the basis of the depth of branching and best-BLAST identities of 91% to sister clade members (see Fig. S1).

**FIG 2:**
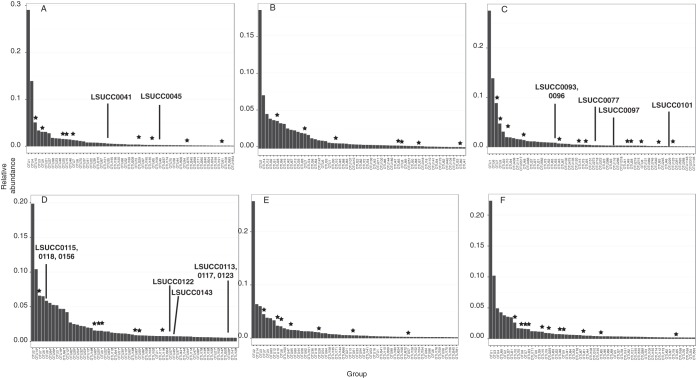
Positions of LSUCC isolates relative to matching OTUs within the top 60 ranks for the first six experiments. OTUs are ordered by decreasing relative abundance, according to the average of duplicate samples. Experiments are in the following order according to Table 2: CJ (A), ARD (B), JLB (C), FWC (D), LB (E), and Tbon (F). The LSUCC isolates with the best BLAST hits to the representative sequence for a given OTU are in bold. Stars indicate OTUs with cultured representatives from other LSUCC experiments. Isolates with matching OTUs with ranks lower than 60 are detailed in Table S1 in the supplemental material.

**FIG 3:**
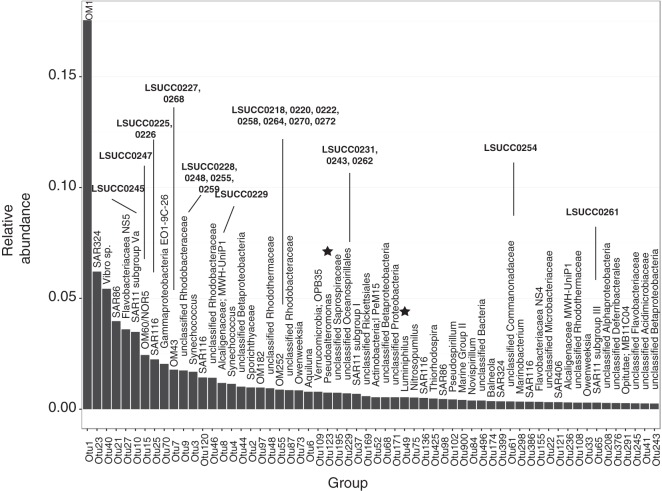
Positions of LSUCC isolates relative to matching OTUs within the top 60 ranks for the most successful experiment, CJ2, which is enlarged to allow for OTU taxonomy labels. OTUs are ordered by decreasing relative abundance, according to the average of duplicate samples. LSUCC isolates with best blast hits to the representative sequence for a given OTU are in bold. Stars indicate OTUs with cultured representatives from other LSUCC experiments. Isolates with matching OTUs with ranks lower than 60 are detailed in Table S1 in the supplemental material.

Comparison of isolate sequences with operational taxonomic units (OTUs) from whole-community sequencing of the source water demonstrated that our method frequently captured some of the most abundant organisms in the system (Fig. 2 and 3; see Table S1 in the supplemental material). For the most successful experiment, designated CJ2, 11 of the 30 isolates cultivated represented 7 of the top 20 OTUs (Fig. 3). Specifically, LSUCC0245/SAR11 subgroup Va was the 6th ranked OTU, while LSUCC0247/OM60/NOR5 clade was 7th, LSUCC0225 and 0226/SAR116 were 8th, and LSUCC0227 and 0268/OM43 were 10th (Fig. 3). Similar results were obtained in the other experiments, although success was varied (Fig. 2; see Table S1 in the supplemental material). Often, if members of the dominant taxa were not recovered in a particular experiment, a representative of that OTU was recovered in a separate experiment. For example, the SAR11 subclade Va isolate LSUCC0245, represented by OTU10, was recovered only in the CJ2 experiment (Fig. 3), but it nevertheless represented the 3rd, 6th, 8th, and 8th ranks in CJ, JLB, LB, and Tbon source waters, respectively (Fig. 2A, C, E, and F). In an even more extreme example, cultivation was totally unsuccessful at LB and ARD, yet isolates from other cultivation attempts represented 5 and 3 of the top 25 OTUs, respectively, in the rank abundance curves from those experiments (Fig. 2B and E, respectively). While the SAR11 subclade Va organism and some other isolates were cultivated only once, several other closely related taxa were obtained more frequently. For example, 10 isolates of the OM252 clade, represented by OTU55, were cultured from three different experiments. This OTU showed various relative abundances (ranks 20 to 540) but regular occurrence, being observed in four of the seven experiments within the top 35 ranks (see Table S1 in the supplemental material). Continued cultivation efforts will provide opportunities for biogeographic analyses of individual isolates, as well as the efficacy and reproducibility of the cultivability of organisms from abundant clades.

Why some experiments were more successful than others can only be speculated upon at this time. There are numerous potential reasons why some organisms have not been isolated at all or why some organisms can be cultivated under some circumstances but not others, even though they are present in the starting inocula. It may have to do with a dependence on an unsupplied compound or combination of compounds, the specific concentration thresholds of compounds, or the requirement for a better surface than PTFE. There is also evidence that many taxa are dormant and can be cultivated after stochastic release from dormancy (28). Additionally, our threshold of 10^4^ cells·ml^−1^ for calling a culture positive may prevent us from obtaining certain taxa that are always at low abundance or those that have not achieved this threshold in the allotted time.

Beginning in March 2015, we attempted to match salinity with measured values obtained with the JW2 to JW4 media by diluting the basic salts and Mg/Ca components (excluding P) (Table 3). Our motivation for making these adaptations was the evidence that salinity is one of the most important determinants of microbial community composition, if not the most important one, in general (29, 30) and in estuarine environments in particular (31–33). The six sites ranged in salinity from 1.4 to 26 ppt (Table 2). The values at CJ, CJ2, and JLB were the most similar to those of marine environments (>22 ppt), while Tbon was brackish (14.6 ppt) and ARD, FWC, and LB nearly qualified as freshwater (<6 ppt) (Table 2). We matched salinity as closely as possible for the FWC, LB, Tbon, and CJ2 experiments (Table 2), but the impact of these efforts was inconclusive. Although there may be a trend emerging across all of our experiments whereby cultivability is correlated with the similarity between *in situ* and medium salinities, as of now, there are too few data points to ascertain a significant pattern.

**TABLE 3:** Comparison of the compositions of natural seawater, marine broth 2216, and JW artificial seawater^a^

Element	Seawater	Marine broth 2216	JW1	JW2	JW3	JW4
Chlorine	19	19.8	19.2	12.8	6.4	3.2
Sodium	10.5	8.8	11	7.4	3.8	2
Magnesium	1.35	2.2	1.27	0.85	0.42	0.211
Sulfur	885	0.72	0.96	0.64	0.32	0.16
Calcium	0.4	0.65	0.41	0.28	0.14	0.07
Potassium	0.38	0.32	0.39	0.26	0.13	0.07
Bromine	0.65	0.054	0.063	0.042	0.022	0.01
Carbon	0.028	4.78	0.0008^b^	0.0008^b^	0.0008^b^	0.0008^b^
Boron	0.0046	0.0047	0.0046	0.003	0.0015	0.00075
Silicon	0.003	0.00085				
Fluorine	0.0013	0.00109	0.001	0.0009	0.00046	0.00023
Nitrogen	0.0005	0.72	0.0096^c^	0.0096^c^	0.0096^c^	0.0096^c^
Phosphorus	0.0007	0.045	0.0016	0.0016	0.0015	0.0008
Iron	0.00001	0.0226	0.000006	0.000006	0.000006	0.000006

a All values are in grams per liter. The data in the seawater and marine broth 2216 columns are from reference 14.

b C as mixed amino acids, fatty acids, carboxylic acids, and sugars (17, 18).

c N as NO_3_^−^, NO_2_^−^, NH_4_^+^, urea, and amino acids (17, 18). Differences between JW media and seawater in some element values are related to the geochemical nature of the Gulf of Mexico compared to the Atlantic subtropical gyre.

We attribute the success of the ASM to keeping constituent concentrations close to environmentally relevant values and providing a complex suite of carbon and nitrogen sources while simultaneously keeping the total organic C level low. However, there are still many taxa that remained uncultivated. Notably, the most dominant members of these communities were *Actinobacteria*, belonging to either the OM1 clade (i.e., “marine *Actinobacteria*” [34]) in marine samples (OTU1; Fig. 2A, C, and F and 3) or the hgcI clade in more freshwater samples (OTU2; Fig. 2B, D, and E). Furthermore, it must be remembered that because of incubation in the dark, this method excluded the cultivation of phototrophs, yet some, like *Synechococcus*, were among the most abundant organisms in some samples (e.g., OTU4; Fig. 2A). Incubation in the light could prove fruitful for the isolation of phototrophs.

We did not attempt to precisely match the concentrations of inorganic nutrients in our media to those at sampled sites during these experiments, but this could also be a key way to improve cultivability. At 5, 2, and 38 µM, the concentrations of ammonium, nitrite, and nitrate, respectively, in our ASM are near the measured values at the sites where we sampled for the cultivation experiments; however, these values fluctuated (see Table S1 in the supplemental material). The nitrate concentration at site CJ was measured at 0.6 µM, while it was measured at 85 µM at ARD and Tbon (see Table S1). Our ASM phosphate concentration (51 µM) was based on previous success with the cultivation of SAR11 (12); however, this was considerably higher than the *in situ* levels (1 to 3 µM) (Table 2; see Table S1). Measurements of other constituent concentrations (e.g., DOM components) could help determine what drives the successful cultivation of more of the microbial majority. Additionally, we expect the physiological and genomic characterization of our isolates to shed additional light on why some taxa may have been preferentially isolated rather than others. On the basis of our observations, future experiments will include additional alterations to the medium to try to obtain additional key members of the microbial consortia.

Nevertheless, while there is always room for improvement, this is the first reported isolation of SAR11, SAR116, OM43, HIMB11-type members of the *Roseobacter* clade, and many other taxa in ASM, which validates the JW medium design and provides a suite of isolates from the Gulf of Mexico that should prove valuable for linking genomics and physiology with biogeography. The modularity of our media also facilitates easy customization for targeted cultivation approaches informed by genomics, metagenomics, or marine chemistry. Furthermore, the success of the ASM recipes in the HTC context fulfills the goal of providing a portable, reproducible cultivation strategy that can be utilized by researchers constrained in their capacity to obtain seawater and thereby provides another valuable tool for the cultivation of important marine clades.

All of the cultures described here are currently archived and available upon request as part of the LSUCC.

## MATERIALS AND METHODS

### Sampling.

One liter of surface water was collected at each site (see Table S1 in the supplemental material) in a sterile, acid-washed polycarbonate bottle (Nalgene). Duplicate 120-ml volumes were filtered serially through 2.7-µm Whatman GF/D and 0.22-µm Sterivex (Millipore) filters, which were immediately stored on ice and transferred to −20°C upon return to the lab until DNA extractions were performed. For biogeochemical measurements, duplicate 50-ml volumes of the 0.22-µm filtrate were collected and placed on ice for measurements of SiO_4_, PO_4_^3−^, NH_4_^+^, NO_3_^−^, and NO_2_^−^ at the University of Washington, Marine Chemistry Laboratory (http://www.ocean.washington.edu/story/Marine+Chemistry+Laboratory). Ten milliliters of whole water for cell counts was fixed with 10% (final volume) formalin and placed on ice immediately. Temperature, pH, dissolved oxygen, and salinity were measured with a handheld YSI 556 multiprobe system.

### HTC experiments.

The remaining surface water was stored on ice and immediately returned to the lab for use in HTC experiments as described by Thrash et al. (15). Briefly, a subsample was filtered through a 2.7-µm GF/D filter, stained with 1× Sybr green (Lonza), and enumerated with a Guava EasyCyte (Millipore) flow cytometer. For each experiment, a total of 480 dilution-to-extinction cultures were established with five 96-well (2.1 ml per well) PTFE plates (Radleys, Essex, United Kingdom) containing artificial media (see Table S1 in the supplemental material). Each well was inoculated with an estimated 1 to 3 cells from the 2.7-µm-filtered water, and plates were stored in the dark at *in situ* temperatures and monitored for growth at 2 to 4 weeks, depending on the temperature, and again at 6 weeks. Growth was monitored with a Guava EasyCyte (Millipore) flow cytometer, and isolates reaching at least 10^4^ cells·ml^−1^ were transferred to 50-ml polycarbonate flasks and cryopreserved in liquid N_2_ with 10% glycerol or 5% (final volume) dimethyl sulfoxide. Normally, all of the isolates reaching this minimum growth were transferred, with one exception, experiment FWC. In that experiment, cells were counted by microscopy (35), and we believe the total cell concentration in our inoculum was underestimated. The resultant cultivability was >53%, and we therefore subselected only 60 wells for transfer. All transferred isolates, upon reaching at least 10^5^ cells·ml^−1^, were collected on 25-mm, 0.22-µm polycarbonate filters (Millipore), and DNA extractions were performed with the MO BIO PowerWater DNA kit in accordance with the manufacturer’s instructions. Additional cryogenic stocks were also made at this stage to increase the number preserved in the LSUCC. Isolate 16S rRNA genes were amplified with recombinant Taq (Invitrogen) and primers 27F/1492R (S-d-Bact-0008-d-S-20/S-*-Univ-1492-a-A-21) (36) under the following PCR conditions: denaturation at 94°C for 30 s, annealing at 50.8°C for 30 s, elongation at 72°C for 2 min, and a final elongation step at 72°C for 10 min; repeated 35 times. Prior to Sanger sequencing, successful PCR products were cleaned with the QIAquick PCR purification kit (Qiagen) to remove any inhibitors and PCR materials. Sanger sequencing of amplicons, with both the forward and reverse primers, was completed at the Michigan State RTSF Genomics Core (https://rtsf.natsci.msu.edu/genomics/). Sanger sequences were automatically curated by the Finch software (Geospiza Finch Suite distribution v 2.21.0) as provided by the Michigan State RTSF Genomic Core. Forward and reverse sequences were assembled, when sufficient overlap permitted, via the CAP3 web server (http://doua.prabi.fr/software/cap3), after conversion of the reverse read to its reverse complement at http://www.bioinformatics.org/sms/rev_comp.html. The purity of isolates was evaluated on the basis of the consistency of the quality scores across the length of the read. All chromatograms are available in the supplemental material with the reference to this report linked to “Supplementary Information.”

### Community iTag analysis.

Community DNA was isolated from the Sterivex filters by removing the filters from their housing under sterile conditions in a biosafety cabinet. Filters were then extracted with the PowerWater kit as well. DNA was sequenced in the 16S rRNA gene V4 region (515F 806R) with Illumina MiSeq 2 × 250-bp paired-end sequencing at Argonne National Laboratories (37). Raw 16S rRNA gene amplicon data were analyzed with mothur v.1.33.3 (38) by using the Silva v119 database (39). Briefly, 16S rRNA sequences were assembled into contigs and discarded if the contig had any ambiguous base pairs, possessed repeats of >8 bp, or were >253 bp long. Contigs were aligned with the Silva rRNA v.119 database, checked for chimeras with UCHIME (40), and classified by using the Silva rRNA v.119 database. Contigs classifying to chloroplast, eukaryotes, mitochondria, or “unknown” affinities were removed from the data, and the remaining contigs were clustered into distinctive OTUs with a 0.03 dissimilarity threshold (OTU_0.03_). Rank abundance curves were arranged by plotting the mean relative abundance of two biological replicates collected and sequenced separately, with only the top 60 OTUs included for clarity. Complete OTU tables are available upon request. Plots were created in R (v. 3.2.1) (41) with the graphing program GGPLOT2 (v. 2.0.0) (42). To determine which OTUs represented LSUCC isolates, sequences from the OTU representative fasta file, provided by mothur with get.Oturep(), was used to create a database file against which the LSUCC isolate 16S rRNA genes could be used in a BLAST search. The commands used are (BLAST v 2.2.26) formatdb -i database.faa -o T -p F and blastall -i queryfile.faa -d database.faa -p blastn -b 2 -v 2 -o outputfile.txt.

All of the best hits ranked at ≥98% identity, with the exception of three sequences that had 96 to 97% matches to very low-ranking OTUs (LSUCC0175, LSUCC0115, and LSUCC0141). The BLAST results (BOR_bestblasthit), OTU rep file (BOR_16S.rep.fasta), OTU taxonomic assignments (BOR_16S.taxonomy), and OTU table (BOR_OTU.csv) are available in the supplemental material with the reference to this report linked to “Supplementary Information.”

### Phylogenetic trees.

Full-length or, if a contig was unavailable, forward 16S rRNA gene sequences from isolates were compiled with best hits from BLAST to the NR database and sequences of known representatives of the various marine clades to which isolates were matching (All fasta files are provided in the supplemental material). Sequences were aligned with MUSCLE (43) and culled with Gblocks (44), and phylogeny was inferred with FastTree2 (45). These processes were linked by using a custom shell script (FT_pipe) that is also available in the supplemental material with the reference to this report linked to “Supplementary Information.” Visualization was performed with Archaeopteryx (46).

### Nucleotide sequence accession numbers.

Community 16S rRNA gene sequence fastq files are available at the NCBI Sequence Read Archive under accession numbers SRR3085688 to SRR3085701. Individual isolate sequences have been submitted to NCBI under accession numbers KU382357 to KU382438.
