# Correction for Henson et al., Artificial Seawater Media Facilitate Cultivating Members of the Microbial Majority from the Gulf of Mexico

**DOI:** 10.1128/mSphere.00124-16

**Published:** 2016-06-01

**Authors:** Michael W. Henson, David M. Pitre, Jessica Lee Weckhorst, V. Celeste Lanclos, Austen T. Webber, J. Cameron Thrash

**Affiliations:** Department of Biological Sciences, Louisiana State University, Baton Rouge, Louisiana, USA

## AUTHOR CORRECTION

Volume 1, no. 2, doi:10.1128/mSphere.00028-16, 2016. Two additional references should have been cited:

**Sosa OA, Gifford SM, Repeta DJ, DeLong EF**. 2015. High molecular weight dissolved organic matter enrichment selects for methylotrophs in dilution to extinction cultures. ISME J **9**:2725–2739. http://dx.doi.org/10.1038/ismej.2015.68.

**Hahnke RL, Bennke CM, Fuchs BM, Mann AJ, Rhiel E, Teeling H, Amann R, Harder J**. 2015. Dilution cultivation of marine heterotrophic bacteria abundant after a spring phytoplankton bloom in the North Sea. Environ Microbiol **17**:3515–3526. http://dx.doi.org/10.1111/1462-2920.12479.

Sosa et al. should be cited on PDF page 1, line 8 of the introduction, after “OM43” (together with reference 7) and also on page 2, line 11, after “components.”

Hahnke et al. should be cited on page 1, line 9 of the introduction, after “others” and also on page 2, line 5, after “concentrations” (together with references 4, 7, 9, 10, and 12).

