# Second Correction for Henson et al., “Artificial Seawater Media Facilitate Cultivating Members of the Microbial Majority from the Gulf of Mexico”

**DOI:** 10.1128/mSphere.00415-18

**Published:** 2018-08-15

**Authors:** Michael W. Henson, David M. Pitre, Jessica Lee Weckhorst, V. Celeste Lanclos, Austen T. Webber, J. Cameron Thrash

**Affiliations:** a Department of Biological Sciences, Louisiana State University, Baton Rouge, Louisiana, USA

## AUTHOR CORRECTION

Volume 1, no. 2, e00028-16, 2016, https://doi.org/10.1128/mSphere.00028-16. We have identified two errors in our 2016 *mSphere* article. The first error occurred in the reported amino acid composition. In Table S1, we listed cysteine × 2 HCl in our amino acid mix, but the correct amino acid was cystine × 2 HCl. While this does not affect the overall rounded organic carbon concentration reported in Table 1, it does affect the organic nitrogen concentration calculation in Table 1 and the total nitrogen concentration reported in Table 3. In addition, we found a second calculation error for organic nitrogen in Table S1, sheet “C and N mixes,” cell O42. These two errors lead us to make the following corrections.

Page 3 of the PDF, Table 1: the concentration for total organic nitrogen should be 17 µM instead of 23 µM.

Page 6 of the PDF, Table 3: total nitrogen should be reported in the JW1 to JW4 columns as 0.00087 g/liter instead of 0.0096 g/liter (and the originally reported value of 0.0096 g/liter should have been 0.00096 g/liter).

Table S1 is updated on our laboratory website, where it is currently hosted (https://thethrashlab.com/publications/).

While the errors described above do not impact the conclusions made in our article, they do inhibit proper reconstruction of the media we used in our experiments. We very much regret the errors.

